# Comprehensive identification of pathogenic tandem repeat expansions in sporadic amyotrophic lateral sclerosis: advantages of long-read vs. short-read sequencing

**DOI:** 10.3389/ebm.2025.10593

**Published:** 2025-07-17

**Authors:** Eleonora Sabetta, Karin Rallmann, Jonas Bergquist, Pille Taba, Abigail L. Pfaff, Bal Hari Poudel, Davide Ferrari, Massimo Locatelli, Sulev Kõks

**Affiliations:** ^1^ IRCCS Ospedale San Raffaele, Milan, Italy; ^2^ Department of Neurology, Tartu University Hospital, Tartu, Estonia; ^3^ Analytical Chemistry and Neurochemistry, Department of Chemistry - Biomedical Center, Uppsala University, Uppsala, Sweden; ^4^ Institute of Clinical Medicine, University Tartu, Tartu, Estonia; ^5^ Perron Institute for Neurological and Translational Science, Perth, WA, Australia; ^6^ Personalised Medicine Center, Murdoch University, Perth, WA, Australia; ^7^ Scienze Chimiche della Vita e della Sostenibilità Ambientale (SCVSA) Department, University of Parma, Parma, Italy

**Keywords:** genetic architecture, sporadic amyotrophic lateral sclerosis (ALS), tandem repeats, neurodegenerative disorders, short-read sequencing, long-read sequencing

## Abstract

Amyotrophic lateral sclerosis (ALS) is a neurodegenerative disorder presenting progressive weakness of the bulbar and extremity muscles, leading to a wide-ranging clinical phenotype. More than 30 genes have been associated to genetically inherited ALS yet, approximately 85%–90% of ALS cases are sporadic. Short tandem repeats expansions, have recently been found in clinically diagnosed ALS patients and are currently investigated as potential genetic biomarkers. In this paper we compare the investigation of pathological tandem repeat expansions on a group of ALS patients by comparing the standard short-read sequencing (SRS) technique with a long-read-sequencing (LRS) method which has recently become more accessible. Blood samples from 47 sporadic ALS cases were subjected to SRS by Illumina Whole Genome Sequencing. The genome-wide tandem repeat expansions were genotyped using GangSTR, while wANNOVAR was used for variant annotation. Uncertain cases were further explored using LRS. SRS identified pathological expansions in *HTT*, *ATXN2*, and *CACNA1A* genes in one patient, which were not confirmed with LRS. The latter identified large tandem repeat expansions in the C9orf72 gene of one patient that were missed by SRS. Our findings suggest that LRS should be preferred to SRS for accurate identification of pathological tandem repeat expansions.

## Impact statement

At present, the pathogenesis of Amyotrophic Lateral Sclerosis (ALS) is not fully understood. Patients may wait as long as one year for a definitive diagnosis, which is still based on clinical criteria. In this regards, the identification of genetic hallmarks would greatly improve the diagnostic path, in particular for sporadic ALS forms. Short tandem repeats (STR) expansions have recently been found in patients with a clinical diagnosis of ALS as potentially causative of the disease and therefore as possible clinical biomarkers. Most of the previous studies identified STR expansions using Short Read Sequencing (SRS). Thanks to technology improvement, Long Read Sequencing (LRS) have recently become more accessible.In this paper, we compared SRS and LRS on a cohort of sALS patients and showed that SRS might fail in identifying pathological repeats as well as misidentify existing pathological repeats.Thus, we believe that our findings will be relevant to the broad readership of your journal, and be of inspiration for future studies that, by including large dataset and the proper sequencing technique, will help elucidating the ALS molecular mechanisms and consequently identify potential therapeutic targets.

## Introduction

Amyotrophic Lateral Sclerosis (ALS) is a neurodegenerative disorder presenting progressive weakness of the bulbar and extremity muscles, leading to a wide-ranging clinical phenotype like Bulbar, Pseudobulbar and Limb ALS, and Limb and Mill’s variant [1–3]. Symptoms onset occurs between 58 and 63 years with a prevalence, among the European population, of 2.2 per 100,000 individuals and a higher incidence in males than in females [4, 5]. Cognitive impairment occurs in up to 50% of cases, with 15% of patients diagnosed with frontotemporal dementia [4]. Approximately 85% of ALS cases are sporadic (sALS) whereas the remaining 10%–15% are familial (fALS) [4, 6], both showing similar clinical presentations [5, 7]. ALS etiology is the result of numerous factors including genetic susceptibility, age-related cellular damage, and environmental exposures among which gender, geographical region, smoking, sportive activities and lead exposure [8, 9].

At present, approximately 30 genes have been associated to ALS [10–12]^.^ Four of them: C9ORF72, SOD1, TARDBP and FUS, account for approximately 60%–70% of familial ALS cases and 6%–10% of sporadic ALS cases, listed in order of decreasing frequency [13]. While known ALS disease genes account for a minority of sporadic cases, recent research highlights the potential role of noncoding structural variants and gene copy number variations in sALS susceptibility and phenotype modification [13]. Interestingly, the pathogenic form of the chromosome 9 open reading frame 72 (C9ORF72) gene is a G4C2 hexanucleotide repeat expansion (HRE) in the intron 1 between the non-coding first exons 1a and 1b [14]. C9ORF72 is currently the only short tandem repeat (STR) expansion proven to cause ALS and frontotemporal spectrum disorder (FTD), however, different expanded STRs distinctive of other neurodegenerative disease like ATXN1 (spinal cerebellar ataxia type 1 (SCA1)), ATXN2 (SCA2), ATXN8 (SCA8) and HTT (Huntington’s disease) [15] have been found in clinically diagnosed ALS patients and FTD cases [16, 17]. Among them, the CAG trinucleotide expansions in ATXN2 have been identified as risk factors for ALS [4, 18]. Interestingly, in addition to the more than 30 genes associated to fALS, heritability studies suggest a 60% genetic component for sALS as well [19] suggesting that sALS is triggered by a complex genetic variation far from being understood.

In this context, STR expansions could represent a valuable starting point to further investigate and clarify the genetic predisposition of sporadic cases as well. Proving the association between STR expansions and sALS will also have beneficial effects on the diagnosis of the disease, which might take up to 1 year, and is still based on clinical, electrophysiological, and radiological investigations while genetic variants or other biomarkers tests are rarely taken into consideration [20].

Next generation sequencing (NGS), like Illumina short-read sequencing techniques (SRS), and the development of various computational methods set the scene for genome-wide STR detection [21]. It has been shown that, due to technical limitations, SRS methods often lack sensitivity and specificity for detecting a significant proportion of structural variants (SVs) and tandem repeats [22]. These limitations can be addressed by long-read sequencing (LRS) which, unlike SRS, can directly sequence long repeat regions without the need for fragmentation. For example, SRS generates reads of 100–150 base pairs, which is much smaller than the thousands of base pairs typical of pathogenic STR expansions. Consequently, SRS is limited in detecting pathogenic STR expansions, but can still identify smaller STRs using specialized tools like GangSTR or ExpansionHunter. In contrast LRS may generate reads up to two megabases (Mb) in length allowing for more efficient detection of larger STR expansions [23]. In this context it is important to note that most reads from LRS platforms (e.g., PacificBiosciences (PacBio), Oxford Nanopore) are typically shorter ranging from 10 to 100 kb for PacBio and 20–200 kb for ONT) [24].

Long read NGS instruments have been on the market for the past decade. Initially, the lower yield, higher error rate, and higher costs of the instruments, have kept them from being more widely adopted. More recently, PacBio (PacBio) and Oxford Nanopore Technologies (ONT) have both been working successfully to make LRS more accessible. These technologies, with the aid of several available computational tools, such as “ExpansionHunter, STRetch, and Tandem Repeat Finder” [21], use information from flanking sequences to provide better alignment for pathogenic STRs. Being a relatively new technique, only a few studies have applied LRS to characterize disease-associated STRs [25–28]. In contrast, most of the previous studies on the characterization of ALS-associated pathogenic STRs were performed using SRS [16, 29, 30].

In our study, we used SRS to sequence the DNA from 47 sALS patients to investigate pathological STR expansion. LRS was then used to reanalyze samples from two patients, as the identification of pathogenic STR expansions raised ambiguities in the interpretation of their STR lengths.

As mentioned above, LRS offers distinct advantages over SRS, including the ability to directly sequence long repeat regions and accurately determine STR sizes, which is crucial for precise quantification of STR expansions.

## Materials and methods

### Population characteristics

Forty-seven patients, accessing the Neurology Department at the University of Tartu between 2013 and 2018, and diagnosed with sALS, based on El Escorial Criteria and the absence of a positive family history were included in the study (Table 1). Blood samples were collected to perform Whole Genome Sequencing (WGS) analysis. The research was conducted with the approval of the University of Tartu Research Ethics Committee (approval: 327/T-L17), and all participants signed a written informed consent.

**TABLE 1:** General characteristics of the 47 ALS patients subjected to WGS. Categorical variables are expressed as absolute count (%), while continuous variables are expressed as median (IQR).

General characteristics of the 47 participants	Median (IQR) or absolute number (Percentage)
Age. years	66 (12.0)
Age at onset. years	64 (14.0)
Positive family history for ALS	0 (0%)
Female	32 (68%)
Ethnicity White	47 (100%)
Duration of diagnosis. months	10 (12.5)
Diagnosis/ clinical subtype of ALS Classic Amyotrophic Lateral Sclerosis Progressive Muscular Atrophy Progressive Bulbar Palsy Primary Lateral Sclerosis	39 (83%) 5 (11%) 2 (4%) 1 (2%)
Symptoms at onset Spinal Bulbar	29 (62%) 18 (38%)

The general characteristics of the population are reported in Table 1. The median age was 65 (interquartile range IQR = 12.5). Most subjects were female (31, 65%) while the remaining 16 (35%) were male. No patient reported a positive family history of ALS; all the participants had a sporadic form. The most frequent clinical subtype was the classic ALS (82%), with spinal symptoms as the most common (61%).

### Short read whole genome sequencing

Library preparation (Illumina DNA preparation kit PCR free) and WGS was performed by the Australian Genome Research Facility (Illumina paired end; 2 × 150bp read length) for all 47 samples. Image analysis was performed in real-time by the NovaSeq 6000 Control Software v1.7.5 while Real-Time Analysis (RTA) v3.4.4. RTA performs real-time base calling on the NovaSeq 6000 instrument computer. The Illumina DRAGEN BCL Convert 07.021.624.3.10.8 pipeline was used to generate the sequence data. The generated FASTQ files were analyzed with FASTQC^1^ to check the quality of the reads. Quality (SLIDINGWINDOW:4:15 LEADING:10 TRAILING:10) and adapter trimming was performed using Trimmomatic 0.38 and reads with a minimum of 36bp were retained [31].

The reads were aligned to the reference genome (hg38; GRCh38_full_analysis_set_plus_decoy_hla.fa) using the Burrow Wheeler aligner (BWA-MEM) [32], converted to a Binary Alignment Map (BAM) file using Sequence Alignment/Map (SAM) tools [33], and duplicates marked using Picard.^2^ The sequencing data was of high quality with an average of 99.8% of reads mapped and 0.12% duplicated reads. The average coverage across the 47 whole genomes was 34x (ranging from 25x to 73x).

### Tandem repeat expansion calling

The bioinformatics tool *GangSTR* ^3^ was utilized to genotype 12 pathogenic STR loci in the human genome [34]. Default settings were used, and quality filtering was performed on the genotypes using dumpSTR.^4^ The following GangSTR-recommended filtering parameters were applied: a minimum call quality of 0.9 and a read depth of at least 20. Genotypes supported only by spanning and/or bounding reads as well as loci where the maximum likelihood genotype estimates were outside the bootstrap confidence interval were excluded. wANNOVAR^5^ was used (reference genome hg38) for variants annotation from our cohort’s Variant Call Format (VCF) [35], using only variants genotypes that passed the quality filter.

### Long-read whole genome sequencing

Oxford Nanopore Technologies (ONT) libraries were constructed using the ligation sequencing kit (SQK-LSK110). Sequencing was performed on an ONT GridION using two flow cells (R9.4.1) for patient 28, super accurate base calling, and a minimum q-score of 10. ONT whole genome sequencing for patient 21, was performed at the Genomics Core Research Facility at Murdoch University using a PromethION. The PromethION enabled increased sequencing output and therefore sequencing depth for improved coverage of the genome (11x vs. 26x). The reads were aligned to the reference genome (GRCh38) using FASTQ files as input and Minimap2 [36]. For these samples, the BAM files were checked manually to compare the calls made by GangSTR on the SRS data.

## Results

Twelve, potentially pathogenic, STR loci located in the following genes: C9orf72, ATXN2, ATXN1, ATXN7, FMR1, DM1-AS, PPP2R2B, ATXN8OS, HTT, CACNA1A, ATXN3, and TBP, were genotyped in all the 47 sALS patients. Out of 564 genotypes, 308 (54.6%) passed quality filtering. Twenty-six genotypes (4.6%) failed level 1 general filters, 25 of these had only spanning and/or bounding reads, and 1 had low read depth. The remaining 230 genotypes (40.8%) failed the more stringent level 2 filter, which enforces a minimum call quality threshold to ensure precise repeat length estimation.

In each individual, on average, 56.4% (0%–92%) of the genotypes at the 12 loci passed filtering. No significant correlation was observed between the average sequencing depth and the number of loci genotyped in each individual (cor = 0.12, p = 0.42). Post filtering, the percentage of genotypes available at each loci ranged from 0% to 83% and there was a significant negative correlation with the number of genotypes called and the size of the repeat in the reference genome (cor = −0.72, p = 0.007). The only locus in which no genotypes passed the quality filtering for the STR located in the TBP gene which was the largest in the reference genome (114bp). More than half of the failed calls were due to the absence of reads fully enclosing the repeat, indicating that longer-read sequencing covering the entire repeat and its flanking regions is necessary for accurate genotyping. This underscores a key limitation of short-read sequencing when genotyping larger genomic repeats. The tandem repeat data are summarized in Table 2.

**TABLE 2:** Tandem repeats genotyped in the population based on WGS SRS data. Categorical variables are expressed as absolute count (%). TBP gene is not displayed.

Genes (locus)	Repeat motif	HGNC ID	Number of patients (%)			Passed QC
			Range			
			Normal	Intermediate	Pathological	
*ATXN7*(3p14.1)	CAG	10560	6 (12.7)	-	-	28
*HTT* (4p16.3)	CAG	4851	14 (29.8)	-	1(2.1)	27
*ATXN2*(12q24.12)	CAG	10555	-	-	1(2.1)	28
*ATXN3*(14q32.12)	CAG	7106	23 (48.9)	1(2.1)	-	27
*CACNA1A* (19p13.13)	CAG	1388	1(2.1)	-	1(2.1)	33
*ATXN1*(6p22.3)	CAG	10548	23 (48.9)	-	-	29
*FMR1*(Xq27.3)	CGG	3775	18 (38.3)	-	-	19
*PPP2R2B* (5q32)	CAG	9305	21 (44.7)	-	-	28
*ATXN8OS* (13q21.33)	CTG/CAG	10561	7 (14.9)	-	-	24
*DM1-AS* (19q13.32)	CTG	53125	-	1(2.1)	-	30
*C9orf72* (9p21.2)	GGGGCC	28337	24 (51.1)	-	4 (8.5)	39

Out of 47 patients, 46 showed tandem repeat lengths within the normal range (Table 2). Only one patient (patient 21) showed pathogenic STR expansions in the HTT (40 CAG length), ATXN2 (36 CAG), and CACNA1A (46 CAG) genes, and intermediate length STR in the ATXN3 (50 CAG) and DM1-antisense RNA (39 CTG) genes. Patient 21 was further investigated by WGS LRS. The sequencing reads from patient 21 were visually inspected in the bam file using Integrative Genomic Viewer (IGV) to determine the repeat lengths, the LRS did not support the STR calls from the SRS data (Table 3).

**TABLE 3:** Comparison of SRS vs. LRS results in patients 21 and 28.

Gene	Patient 21		Patient 28	
	SRS	LRS	SRS	LRS
ATXN7	N/A	10/10	N/A	10/12
HTT	18/59	18/18	N/A	18/19
ATXN2	22/59	22/22	N/A	21/22
ATXN3	8/58	8/16	N/A	17/21
CACNA1A	8/59	8/14	N/A	4/7
ATXN1	29/60	29/32	N/A	27/29
FMR1	20/36	20/36	N/A	29/31
PPP2R2B	N/A	10/13	N/A	10/14
ATXN8OS	N/A	9/13	N/A	7/15
DM1-AS	5/59	5/5	N/A	5/5
C9orf72	2/6	2/6	N/A	**10/1050**

The table describes the number of repeats called at each loci by GangSTR SRS or from manual inspection of the LRS. Pathogenic repeat expansions are in bold. N/A: not available.

When analyzing the C9orf72 intron 1, located between the non-coding first exons 1a and 1b, for potentially pathogenic hexanucleotide repeat expansion (HRE), the SRS did not reveal any allele neither in the intermediate nor in pathological length range. However, inspection of the calls made by GangSTR for the C9orf72 repeats, prior to filtering, suggested that two samples (patients 28 and 29) might carry pathogenic expansions at this locus. Such calls were filtered out due to low quality.

**FIGURE 1:**
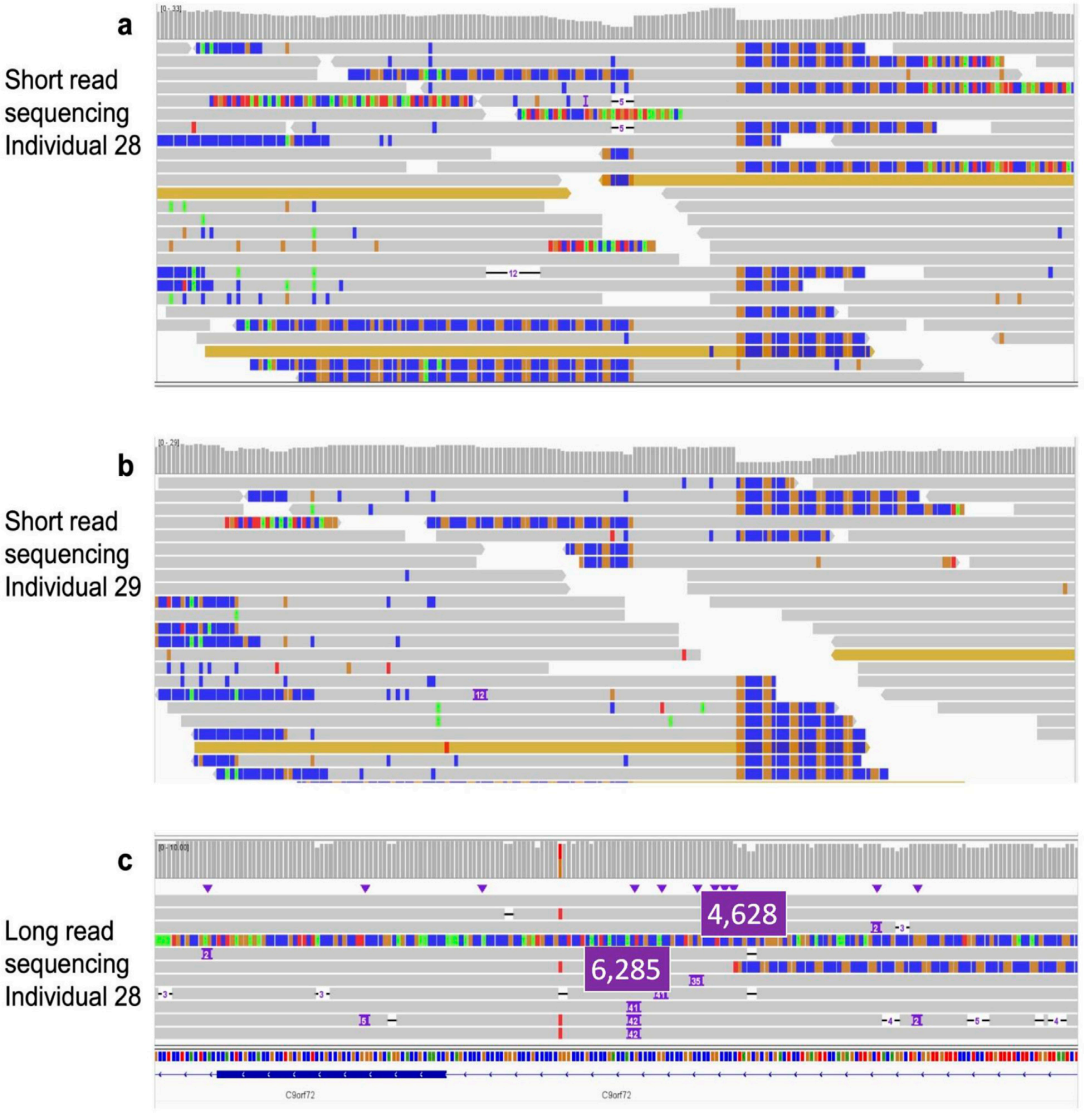
Panel **(a)**: SRS reads over the repeat visualized in IGV for patient 28. Panel **(b)**: SRS reads over the repeat visualized in IGV for patient 29. Panel **(c)**: LRS for patient 28. For graphical reason, the number of the pathological tandem repeats have been highlighted (panel **c**).

Moreover, the number of soft clipped reads observed over the C9orf72 loci (Figures 1a,b) also suggests the presence of STR expansions for both patient 28 and 29.

LRS was then performed for patient 28 only because the DNA available for patient 29 was insufficient for long-read library construction (unfortunately patient 29 died before obtaining the SRS data). LRS for patient 28 (Figure 1c) showed two reads over the C9orf72 hexanucleotide repeat containing large expansions: the first one containing an additional 4628 bp (771.3 repeats) and the second one containing an additional 6285 bp (1047.5 repeats), confirming the presence of the pathogenic C9orf72 STR expansion in the genome of patient 28 (Table 3). From a pathological standpoint, patient 28 exhibited, in addition to motor neuron disease symptoms, a dementia syndrome suggestive of frontotemporal dementia. The disease progression was very rapid. In contrast, patient 29 followed a typical ALS course, beginning with bulbar paralysis followed by the emergence of additional typical ALS symptoms.

## Discussion

ALS is a neurodegenerative disorder presenting phenotypic and genetic heterogeneity [37] with a multifaceted molecular basis difficult to characterize. The identification of reliable biomarkers could positively impact the understanding of the underlying disease mechanisms and, consequently, patient diagnosis and management. In this context, Feldman et al. [4] recently proposed to replace the categorization of sALS and fALS cases with a new binomial: genetically vs. non-genetically confirmed forms, respectively, underlying the importance of genetic testing in disease characterization. Despite the approximately 30 genes already associated to ALS [10–12] detecting, genetically, sALS forms is challenging because they display a clear genetic background in a minority of patients only [38]. Furthermore, the challenge increases when STR expansions are involved as predisposing mutations. For instance, no consensus on a specific disease-related threshold for various polyglutamine-associated disorders has been reached, since healthy individuals may also carry expansions in the pathological range [39]. Although C9orf72 expansions have been extensively associated with ALS/FTD, a disease-causing cut-off for the hexanucleotide repeats is still questioned [40]. Both healthy and affected individuals show repeats in the intermediate range (20-30 hexanucleotides), confirming that the pathological role of intermediate STR expansions is far from being understood [4, 41–45]. Furthermore, with the exception of C9orf72, the abundance of STR expansions in ALS patients compared to healthy control subjects is often narrow [30, 46]. For these reasons, the presence of STR expansions must be evaluated with a robust and reliable sequencing technique, displaying both high diagnostic sensitivity and specificity. Our study showed that even very long STR expansion might not be properly identified by SRS and, on the other end, SRS can falsely identify STR expansion not present in the subject genome. This could result in false negatives, delaying ALS diagnosis and hindering timely clinical management, access to disease-modifying therapies, multidisciplinary care, and clinical trial participation [47]. Although less common, false positive could lead to unnecessary and potentially anxiety-inducing tests.

Thus, LRS must be the preferred choice genotyping a patient DNA in search of pathological STR expansions.

From an epidemiological point of view, despite the limitation due to the relatively small patients cohort and the recruitment from a single Neurology Department, our study seems consistent with previous ones showing a 5–10% of sALS patients carrying C9orf72 STR expansion [16]. Because patient 29 showed SRS characteristics similar to patient 28, we might speculate that both carried pathological STR expansion. However, because no DNA was available for LRS, our hypothesis remains a conjecture. Thus, 4–5% of our sALS cohort showed the presence of expanded C9orf72 hexanucleotide repeats.

## Conclusion

Our study highlighted the benefits of LRS for accurate characterization of large tandem repeats: SRS identified multiple REs in a patient which were not confirmed by long read sequencing. Conversely, in another patient, unfiltered calls from GangSTR (that did not pass the quality filtering) as well as manual inspection of the bam files suggested the presence of expanded alleles in C9orf72 which were further confirmed by LRS. This could lead to ALS misdiagnosis, resulting in either false negatives or false positives, along with the various problems associated with these types of medical errors. These point out SRS limitations in evaluating broader repeat sequences and large genomic rearrangements (32) and recommend the use of LRS to flank ALS clinical diagnosis [48].

## Data Availability

The original contributions presented in the study are included in the article/Supplementary Material, further inquiries can be directed to the corresponding author.

## Footnotes

1
https://www.bioinformatics.babraham.ac.uk/projects/fastqc/

2
http://broadinstitute.github.io/picard/

3
https://github.com/gymreklab/GangSTR

4
https://trtools.readthedocs.io/en/stable/source/dumpSTR.html

5
https://wannovar.wglab.org/
